# Serum Magnesium after Kidney Transplantation: A Systematic Review

**DOI:** 10.3390/nu10060729

**Published:** 2018-06-06

**Authors:** Anne-Sophie Garnier, Agnès Duveau, Martin Planchais, Jean-François Subra, Johnny Sayegh, Jean-François Augusto

**Affiliations:** 1 LUNAM Université, 49180 Angers, France; AnneSophie.Garnier@chu-angers.fr (A.-S.G.); agnes.duveau@chu-angers.fr (A.D.); martin.planchais@chu-angers.fr (M.P.); jfsubra@chu-angers.fr (J.-F.S.); josayegh@chu-angers.fr (J.S.); 2 Université Angers, CHU Angers, Service de Néphrologie-Dialyse-Transplantation, CHU d’Angers, 49933 Angers CEDEX 9, France; 3 CRCINA, INSERM, Université de Nantes, Université d’Angers, LabEx IGO Immunotherapy, Graft, Oncology, 44007 Angers, France

**Keywords:** Magnesium-Kidney, transplantation-New-onset, diabetes after transplantation-cardiovascular risk

## Abstract

Magnesium (Mg) status has recently drawn close attention in chronic kidney disease and in kidney transplant recipients. This review aims to evaluate the body of evidence linking hypomagnesemia to clinical consequences in these specific populations. After a brief summary of the main mechanisms involved in Mg regulation and of Mg status in end-stage renal disease, the review focuses on the relationship between hypomagnesemia and cardiovascular risk in kidney transplant recipients. A body of evidence in recent studies points to a negative impact of hypomagnesemia on post-transplant diabetes mellitus (PTDM) and cardiovascular risk, which currently represent the main threat for morbidity and mortality in kidney transplantation. Deleterious biological mechanisms induced by hypomagnesemia are also discussed. While data analysis enables us to conclude that hypomagnesemia is linked to the development of PTDM, studies prospectively evaluating the impact of hypomagnesemia correction after kidney transplantation are still lacking and needed.

## 1. Magnesium: Physiology

Magnesium (Mg) is the fourth cation of the body and the second most prevalent intracellular cation [1]. Approximately half of total body Mg is located in bone [2], the remainder being contained in skeletal muscles and soft tissues [1]. Extracellular Mg represents only 1% of total body Mg [3] and is mostly found in serum with concentrations ranging between 0.65 to 1.05 mmol/L [4] and in red blood cells [3]. It is present in three different states: ionized Mg (55–70%), protein-bound Mg (20–30%), and Mg complexed with anions such as bicarbonate or phosphate (5–15%) [1].

Mg homeostasis is mainly dependent on intestinal absorption and renal excretion. In the intestine, absorption is modulated by luminal Mg concentration, at high concentrations, Mg is regulated by an active transcellular transport and passive paracellular diffusion; whereas in low concentrations, Mg is absorbed by an active transcellular pathway involving a Transient Receptor Potential Melastatin 6 (TRPM6) channel expressed on the small intestine cells [5].

**Figure 1:**
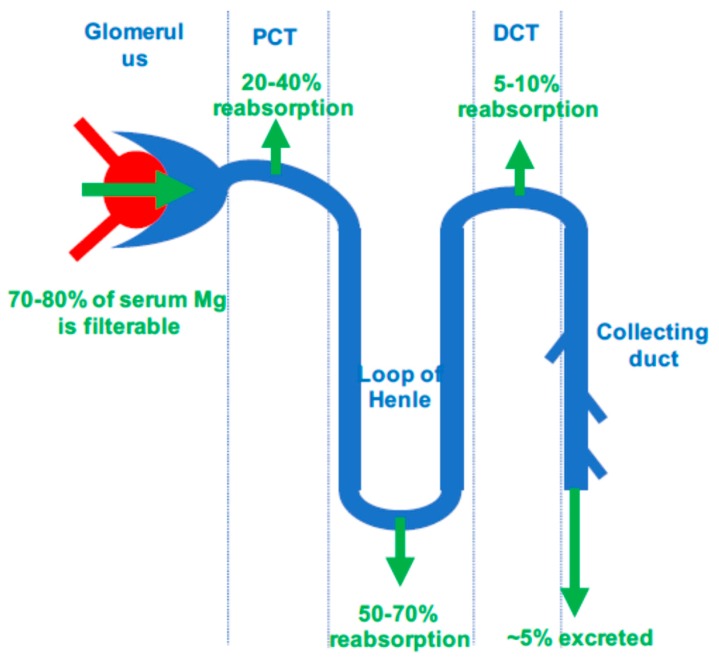
Magnesium exchanges in nephron. PCT, proximal convoluted tubule; DCT, distal convoluted tubule.

In patients with normal kidney function, around 70 to 80% of plasma Mg is ultra-filtrable. About 40% of the filtered Mg is reabsorbed in the proximal tubule by paracellular uptake, while 50% is absorbed in the ascending limb of the loop of Henle and 5% is absorbed in the distal tubule by an active transport. Thus, only 5% of filtered Mg is excreted in final urine, but this excretion can vary widely from 0.5 to 70% to keep plasma Mg concentration within the normal range [6]. Figure 1 summarizes Mg exchange in nephron.

Hypomagnesemia is frequently associated with hypokalemia [7]. This is mainly due to common disorders leading to ion wasting, such as through diuretic intake or diarrhea.

However, reduction of intracellular Mg concentration induces a decline in ATP activity that in turn affects potassium ATP-dependent channels located in the ascending limb and cortical collecting tubule, leading to urinary potassium loss [8,9]. This mechanism may explain the common resistance to hypokalemia correction until hypomagnesemia is normalized.

## 2. Clinical Signs and Etiologies of Hypomagnesemia

Clinical manifestations of hypomagnesemia are quite unspecific. Early signs include nausea, vomiting, anorexia and weakness [1]. Neuromuscular signs can also be present, including numbness, tingling, cramps, fasciculation seizures and neuropsychological disorders [1]. Severe hypomagnesemia has been associated with cardiac arrhythmia and coronary spasm [10]. Mg depletion can induce changes in electrocardiogram readings from pointed T waves in mild hypomagnesemia to widening of the QRS complex, prolongation of the PR interval and ventricular arrhythmias in patients with severe Mg depletion. Arrhythmias are more frequently observed in the setting of an acute ischemic event [11], congestive heart failure—partly due to the administration of diuretics—or after a cardiopulmonary bypass [12]. Some authors showed that administration of magnesium after a cardiopulmonary bypass may prevent postoperative arrhythmias [13].

Hypomagnesemia results mainly from gastrointestinal and renal losses. Gastrointestinal losses can arise from diarrhea, malabsorption, steatorrhea, small bowel bypass surgery, acute pancreatitis and dietary deprivation [14]. Rarely, hypomagnesemia may occur due to a selective defect in the intestinal Mg absorption, also known as primary intestinal hypomagnesemia. Urinary Mg losses can be induced by loop or thiazide-type diuretics, volume expansion, alcohol, hypercalcemia, nephrotoxins such as aminoglycoside antibiotics, cisplatin or cyclosporine. Urinary Mg wasting can also be due to loop of Henle or distal tubule dysfunction, after acute tubular necrosis, post-obstructive diuresis or kidney transplantation [1,14]. Lastly, primary renal Mg wasting was observed in Bartter and Gitelman syndromes, in which hypomagnesemia is associated with hypercalciuria and hypocalciuria respectively [14].

## 3. Magnesium Status in CKD Patients

Chronic kidney disease (CKD) is frequently associated with hypermagnesaemia, which is usually mild or asymptomatic until end-stage renal disease (ESRD). In moderate CKD patients, increased fractional excretion of Mg compensates for a decline in renal function, providing a stable serum Mg within the normal range. However, in CKD stages IV-V, this compensatory mechanism becomes insufficient which results in hypermagnesaemia [6]. Thus, in this setting, dietary Mg intake and administration of Mg-containing drugs, such as laxatives or antacids, can more easily induce hypermagnesaemia [6]. Alternatively, Mg balance may be negative in some ESRD patients on high doses of diuretics, with reduced gastrointestinal uptake induced by severe metabolic acidosis or reduced albumin levels [5].

Dialysate Mg concentration has a significant impact on Mg balance in hemodialysis (HD) or peritoneal dialysis (PD) patients. Indeed, Mg has a significant diffusion across dialysis membranes and its elimination depends on ultrafiltration and the diffusible gradient between the serum and dialysate concentration. A significant reduction in serum Mg concentration has been observed in HD patients, probably due to the Gibbs–Donnan effect [15]. Conversely, mild hypermagnesemia is frequently observed in PD patients, because Mg concentration of PD “standard dialysate” is about 0.75 mmol/L [16]. Interestingly, bone appears as the greatest Mg reservoir in CKD patients. To support this, a 70% increase in both cortical and trabecular bone Mg content has been reported in uremic patients, compared to non-uremic patients, suggesting that dialysis induces positive net Mg balance in ESRD patients.

## 4. Magnesium and Cardiovascular Risk in CKD Patients

Several studies have shown an association between Mg status and survival in ESRD patients. Indeed, Ishimura et al. investigated the prognostic value of serum Mg concentration in 515 patients on maintenance hemodialysis (60 ± 12 years, 306 males and 209 females; 24% diabetics). Mortality was significantly higher in the lower Mg group (<2.77 mg/dL, i.e., <1.14 mmol/L, *n* = 261), compared to the higher Mg group (≥2.77 mg/dL, *n* = 254) (*p* < 0.001). Thus, serum Mg was predictive of mortality (HR (per 1 mg/dL increase), 0.485 (95% CI, 0.241–0.975), *p* = 0.0424), particularly of non-cardiovascular mortality (HR 0.318 (95% CI, 0.132 to 0.769), *p* = 0.0110), after adjustment on confounding factors, including age, gender, hemodialysis duration and presence of diabetes [17]. Likewise, in a nationwide registry-based cohort of 142,555 hemodialysis patients, Sakaguchi et al. moreover observed a U-shaped relation with higher all-cause and cardiovascular mortality of patients in both the lowest Mg sextile (<0.95 mmol/L) and the highest (>1.27 mmol/L) [18].

Several studies maintain that the increased cardiovascular mortality in hypomagnaesemic ESRD patients may be related to accelerated atherosclerosis. In an observational study, PD patients who developed arterial calcifications had significantly lower serum Mg levels (*p* < 0.001) [19]. Similar results were found in a retrospective cohort of 390 non-diabetic and hemodialysis patients. Serum Mg was significantly lower in patients with vascular calcification than in those without (2.69 ± 0.28 vs. 2.78 ± 0.33 mg/dL, *p* < 0.05). Serum Mg concentration appeared as an independent risk factor of vascular calcification (OR 0.28, 95% CI 0.09–0.92/1 mg/dL increase in serum magnesium, *p* = 0.036) after adjustments for age, gender, duration of hemodialysis, calcium, phosphate and intact parathyroid hormone concentrations [20]. Given these observations, some authors investigated the effect of Mg supplementation in ESRD patients. In one study, 47 hemodialysis patients were randomized to one group receiving oral Mg citrate (610 mg per day) and oral calcium acetate, and the other oral calcium acetate and a placebo. After 2 months, patients receiving Mg had a significant decrease in intima-media thickness (0.70 vs. 0.97 mm, *p* = 0.001 and 0.78 vs. 0.95 mm, *p* = 0.002 for left and right carotid arteries respectively) [21]. In another work, hemodialysis patients were randomized to receive low (0.5 mmol/L) or high (0.75 mmol/L) dialysate Mg and were followed-up for 3 years. No difference was observed for all-cause mortality between groups, but an increase in cardiovascular mortality was observed after 3 years in the low dialysate Mg group (14.5% vs. 0%, *p* = 0.042) in HDM group [22].

## 5. Magnesium Status after Kidney Transplantation and Relation with Graft Function

Hypomagnesemia is frequently observed after kidney transplantation, in part to immunosuppressive regimens including calcineurin inhibitors (CNI) that induce Mg urinary waste. Hypomagnesemia was observed in 6.6% of patients treated with tacrolimus and in 1.5% of patients on cyclosporine [23]. The mechanisms leading to hypomagnesemia are not fully understood, but it has been shown that CNI induce a down-regulation of renal expression of the epidermal growth factor [24] and TRMP6 in the distal collecting tubule [25], leading to decreased Mg reabsorption. Sirolimus might induce hypomagnesemia through inhibition of Na-K-Cl co-transporter 2 expression in the thick ascending loop of Henle [26]. Renal Mg wasting has been shown to be similar between rats treated with sirolimus and those treated with cyclosporine or tacrolimus [27]. Many other factors influence Mg levels after kidney transplantation, such as post-transplantation volume expansion, metabolic acidosis, insulin resistance, decreased gastro-intestinal absorption due to diarrhea, low Mg intake and medication such as diuretics or proton pump inhibitors [28].

Hypomagnesemia was reported to develop frequently within the first few weeks following transplantation [29], with a serum Mg level nadir in the second month post-transplantation [30]. Hypomagnesemia may persist for several years after kidney transplantation. In a cohort of 49 kidney transplant recipients, 22.4% of patients had hypomagnesemia 6 years after transplantation [31]. As observed in the general population, serum Mg levels were inversely correlated with glomerular filtration rate [32].

The relationship between serum Mg and graft function has been poorly evaluated in literature. In a cohort study published in 2005, 320 kidney recipients were divided into two groups, based on median Mg level in the entire cohort: the low serum Mg group (*n* = 29, 0.74 (0.68–0.78) mmol/L) compared to the normal Mg group (*n* = 31, 0.9 (0.82–0.98) mmol/L, *p* < 0.05). The authors showed that hypomagnesemia was associated with a greater decline in allograft function and an increased risk of graft loss for patients with ciclosporine-induced nephropathy [33]. In animals studies, hypomagnesemia is associated with glomerular dysfunction and the development of chronic fibrotic lesions [34]. In mice treated with cyclosporine, Mg supplementation improved renal function and decreased kidney fibrotic lesions [35]. Likewise, Mg supplementation in cyclosporine-treated rats was associated with a reduction in tubular atrophy and interstitial fibrosis and prevented kidney function decline [34].

In murine studies, Mg supplementation has been shown to exert an effect using several mechanisms, including innate immune pathways. Indeed, Mg supplementation inhibits monocyte and macrophage recruitment, partly by abolishing expression of chemoattractant proteins (osteopontin and monocyte chemoattractant protein-1), fibrogenic molecules and extracellular matrix proteins [36]. Moreover, Mg induces down-regulation of endothelin-1 expression [36] and decreased nuclear factor kappa-light-chain-enhancer of activated B cells (NFkB) activation [37].

These data in human and mice converge to suggest that hypomagnesemia is not only associated with accelerated decline of graft function but is also an active contributor to renal lesions. Thus, prospective studies are needed to analyze the potential benefits of Mg supplements on graft function after kidney transplantation.

## 6. Serum Magnesium and New-Onset Diabetes Mellitus after Transplantation

### 6.1. Serum Magnesium and Diabetes Mellitus in the General Population

Hypomagnesemia has been reported to occur in 13.5 to 47.7% of non-hospitalized patients with type-2 diabetes [38]. Poor dietary Mg intake, glomerular hyperfiltration, osmotic diuresis, recurrent metabolic acidosis, hypophosphataemia and hypokalemia are all potential contributing factors for hypomagnesemia in diabetic patients [38,39]. A higher incidence of hypomagnesemia was reported in females as compared to males on a 2-to-1 ratio [40]. Several authors showed an inverse correlation between Mg intake and the risk of developing diabetes mellitus [41,42,43,44]. Moreover, in a study including 39,345 US women followed-up for 6 years, the protective role of high Mg intake was higher in the subgroup of overweight women [42]. In the Atherosclerosis Risk in Communities Study, low serum Mg level was an independent risk factor for incident diabetes mellitus [44].

The diabetogenic effects of hypomagnesemia are not yet well understood and have been attributed to several mechanisms. Data supporting the fact that hypomagnesemia may induce altered cellular glucose transport, reduced pancreatic insulin secretion, defective post-receptor insulin signaling and/or altered insulin–insulin receptor interactions have been reported. Dietary-induced Mg deficiency increased urinary thromboxane concentration and enhanced angiotensin-induced aldosterone synthesis, resulting in resistance to the effect of insulin [45]. This may be due to changes of tyrosine kinase expression on insulin receptor level and/or induction of inflammation and oxidative stress [46]. Conversely, it is interesting to note that oral Mg supplementation during a 16-week period showed an improvement in insulin sensitivity and a better metabolic control in type-2 diabetic patients [47].

### 6.2. Serum Magnesium and New-Onset Diabetes Mellitus after Transplantation

After transplantation, diabetes mellitus is frequently observed, with incidences ranging from 10 to 30%, depending on the criteria used for diagnosis and the length of follow-up [48,49]. Post-transplant diabetes mellitus (PTDM) affects both patient and graft survival [50], highlighting the importance of identifying potentially modifiable risk factors. Several risk factors for PTDM have already been identified, such as older age, male gender, ethnicity, acute rejection, hepatitis C, higher body mass index, higher pre-transplant glucose levels and higher trough tacrolimus levels [51,52,53], some of them being common risk factors for type-2 diabetes in the general population. Hypomagnesemia has recently been identified as an independent risk factor for PTDM, however with some conflicting data in literature. Van Laecke et al., in a retrospective study, were the first to identify a relationship between PTDM and post-transplant Mg level in a cohort of 254 kidney transplant recipients. Serum Mg values were recorded at months 1 and 2 after transplantation. 29.5% of recipients developed PTDM after a mean time of 90 ± 80 days post-transplantation. Patients with PTDM had significantly lower Mg levels compared to those without (*p* < 0.001). Post-transplant Mg appeared as an independent predictor of PTDM after adjustment on classical risk factors and on CNI use. Moreover, the association between the use of CNI and PTDM disappeared after adjustments to Mg levels in the multivariate analysis, suggesting that the diabetogenic effect of CNI may be more related to hypomagnesemia itself than to CNI [53]. In another study recently published, the association between serum Mg level and PTDM was examined in a retrospective cohort study of 948 non-diabetic kidney transplant recipients. The authors used multivariable Cox proportional hazards models to evaluate the risk of PTDM as a function of baseline (at 1 month post-transplantation), time-varying (every 3 months) and rolling-average (mean for 3 months moving at 3-month intervals). Hypomagnesemia, defined as a serum Mg concentration below 0.74 mmol/L, was significantly associated with an increased risk of PTDM in baseline (HR, 1.58; 95% CI, 1.07 to 2.34; *p* = 0.02), time-varying (HR, 1.78; 95% CI, 1.29 to 2.45; *p* = 0.001) and rolling-average models (HR, 1.83; 95% CI, 1.30 to 2.57; *p* = 0.001) [54].

The association between hypomagnesemia and PTDM was also studied in pediatric kidney recipients. In a retrospective cohort of 173 young recipients with a median age of 7 years at transplantation, 20 patients developed PTDM at 9 days post transplantation on average. Hypomagnesemia and high tacrolimus levels were significant and independent risk factors for PTDM (*p* = 0.01 and *p* < 0.001, respectively). No association between hypomagnesemia and tacrolimus levels was observed, suggesting that both risk factors were independent from each other [55]. Conversely, in a cohort study of 451 pediatric solid organ transplant recipients, Chanchlani et al. failed to identify an association between hypomagnesemia and PTDM. However, lack of close Mg monitoring and frequent Mg supplementation in their cohort (75% of children) make the study difficult to interpret [56]. Two other studies failed to demonstrate a significant difference in Mg levels between PTDM and non-PTDM adult recipients [57,58]. Osorio et al. analyzed 589 kidney recipients and did not identify a relationship between Mg levels and occurrence of PTDM in patients receiving CNI treatment [57]. Santos et al. observed similar results in a cohort of 205 kidney recipients [58]. However, again, in these studies, no information was given about immunosuppressive regimens, which also limits their interpretation.

In order to minimize the possible effect of post-transplant confounders, our group evaluated the relationship between pre-transplant hypomagnesemia and the risk of PTDM in a cohort of 154 kidney transplant recipients. 28 patients (18.2%) developed a PTDM within the first year of transplantation, and in most patients within the first 2 months. Patients who developed PTDM were older, had a higher body mass index and a higher pre-transplant glucose level, compared to patients without PTDM. The pre-transplant Mg level was significantly lower in patients that developed PTDM (*p* = 0.014). Moreover, a pre-transplant Mg level <2 mg/dL compared to >2.3 mg/dL was associated with a higher risk of PTDM within the first year of transplantation (HR, 2.99; 95% CI, 1.07–8.37, *p* = 0.037) [49]. In the field of liver transplantation, a retrospective cohort of 169 patients showed that both pre-transplant and month 1 post-transplant Mg levels were independent risk factors for PTDM [59]. In these studies, patients were free of diabetes mellitus at inclusion, thus hypomagnesemia was not the result of Mg wasting induced by diabetes.

Based on these observations, Van Laecke et al. investigated the effect of Mg supplementation on post-transplant glucose metabolism [60,61]. In a single-center parallel group study, 54 patients with serum Mg level ≤1.7 mg/dL were randomized to receive 450 mg oral Mg oxide (MgO) one to three times daily, aiming for an Mg concentration of >1.9 mg/dL (*n* = 27) or no treatment (*n* = 27). The primary outcome was a fasting serum glucose concentration at 3 months post-transplantation. Secondary outcomes were the 2 h area under the curve (AUC) for glycaemia and insulin resistance, assessed by a homeostasis model assessment-estimated insulin-resistance index (HOMA_IR) at month 3 post-transplantation. Six patients in the control group received MgO (450 mg daily) as serum Mg concentration dropped below to 1.2 mg/dL. Fasting serum glucose concentration was lower in the Mg group compared to the control group (95% CI; 1.7–21.3; *p* = 0.02), even after adjustment on tacrolimus concentrations. No differences were observed between groups for 2 h-AUC glucose and HOMA-IR. The authors suggest that a disparity in the timing of supplementation and consequent drug exposure along with the use of tacrolimus versus cyclosporine can explain the smaller effect of their intervention compared with other trials [60]. Similar results were observed in another study including 52 renal transplant recipients on tacrolimus with chronic hypomagnesemia. Recipients were randomized to the Mg group (*n* = 26), with a similar Mg supplementation as previously described, or the control group (*n* = 26). No differences between the groups were observed in first-phase insulin release, in second-phase insulin release, HbA1c and HOMA-IR index at month 6 post-transplantation [61]. The main limitation of this study was that oral Mg supplementation failed to increase both serum and intracellular Mg significantly over the concentrations observed in the control group. Thus, it was impossible to draw conclusions on the effects of Mg supplementation on insulin resistance and glucose metabolism.

While most studies converge to confirm that hypomagnesemia is an independent risk factor for PTDM, the impact of hypomagnesemia correction after kidney transplantation has not yet been fully explored. Literature analysis also highlights the need for studies in order to determine the best routes for Mg supplementation, formulations and doses to achieve normal serum Mg concentration in these patients.

An important issue is the impact of immunosuppressive regimen according to diabetes risk and their impact on post-transplant Mg status. As underline above, CNI induce renal Mg wasting which contributes to hypomagnesemia. In the study from Van Laecke et al., the association between CNI and PTDM disappeared after adjustment on Mg levels, suggesting that the diabetogenic effect of CNI was at least partially related to CNI-induced hypomagnesemia [53]. The switch from CNI to mTOR inhibitors has been shown to result in serum Mg increase [62]. Thus, the early use of mTOR inhibitors soon after transplantation could be an interesting approach to decrease PTDM risk. However, several reports have shown a diabetogenic effect of mTOR inhibitors, and in the study from Van Laecke et al., sirolimus appeared as an independent risk factor of PTDM [53,63]. Moreover, the use of mTOR inhibitors soon after kidney transplantation has been shown to be associated with a higher risk of allograft rejection [64]. For these reasons, mTOR inhibitor in replacement of CNI may not be an interesting approach to reduce PTDM risk. 

## 7. Magnesium Status and Cardiovascular Risk before and after Kidney Transplantation

PTDM-associated mortality is mainly related to cardiovascular events, which are today the main causes of death in kidney transplant patients [65]. Hypomagnesemia has been shown to play a role in the pathogenesis of arterial hypertension, endothelial dysfunction, dyslipidemia and inflammation, with all these factors contributing to coronary heart disease (CHD).

In vitro, exposure of endothelial cells to low Mg concentrations reversibly inhibits endothelial proliferation and was associated with an up-regulation of interleukin-1, Vascular Cell Adhesion Molecule-1 and Plasminogen Activator Inhibitor-1 [66]. In vivo, hypomagnesemia is associated with increased CRP levels, leukocyte and macrophage activation, NFKB/cytokines activation and platelet aggregation. Furthermore, inbred mice with low intracellular Mg levels have significantly impaired endothelial function together with decreased endothelial NO synthase expression [67].

The relationship between CHD and serum Mg concentrations was studied in a cohort of 13,922 middle-age adults. In this study, after adjustment, the relative risk of CHD across quartiles of serum Mg was 1.00, 0.92, 0.48, and 0.44 (*p* for trend = 0.009) among women and 1.00, 1.32, 0.95, and 0.73 (*p* for trend = 0.07) among men. Moreover, patients who developed CHD had a lower serum Mg concentration than the controls, suggesting that low serum Mg was an independent risk factor for CHD [68].

In a Japanese cohort of 728 subjects, lower serum Mg was significantly and independently associated with mean intima-media thickness (*p* = 0.004) and risk of ≥2 carotid plaques (*p* = 0.03) [69]. Hypomagnesemia was also reported to directly or indirectly affect vascular stiffness in the general population [70]. In another study, Mg supplementation improved endothelial dysfunction in patients with CHD [71]. In parallel, hypomagnesemia may play a role in the promotion and progression of vascular calcification as underlined earlier. Indeed, Mg is known to prevent tissue calcification by increasing natural inhibitors of calcification such as fetuin A, carboxylated matrix Gla protein (MGP), osteopontin and the inorganic inhibitory compound pyrophosphate [72].

Few studies investigated the association between Mg levels and cardiovascular risk after kidney transplantation. In a small crossover trial published in 1998, 15 renal transplant patients were randomized in a 6-week treatment period with either placebo or Mg oxide (MgO) 2 g per os with a 2-week washout interval, then 6-weeks of the alternative agent (placebo or MgO). There was no reduction in systolic or diastolic blood pressure in either cholesterol or triglyceride level with magnesium supplementation [73].

Finally, the association between serum Mg level and cardiovascular risk after kidney transplantation has been poorly studied. A small crossover trial, including 15 kidney transplant patients randomized in a 6-week treatment period with either the placebo or Mg oxide (MgO) 2 g per os with a 2-week washout interval, then 6-weeks of the alternative agent (placebo or MgO), was published 1998. No change in metabolic profile, including systolic or diastolic blood pressure control, or cholesterol or triglyceride levels was observed with Mg supplementation [73]. The relationship between hypomagnesemia and vascular stiffness was investigated in a study published in 2011. An evaluation was conducted in 512 renal transplant recipients, by determination of carotid-femoral pulse wave velocity. Serum Mg was an independent risk factor for arterial stiffness, but this association was attenuated after adjustment on the use of sirolimus (*p* = 0.054). After stratification according to the median age of 55 years and adjustment with covariates, Mg remained an independent predictor of pulse wave velocity (*p* = 0.024) [74].

## 8. Conclusions

**Figure 2:**
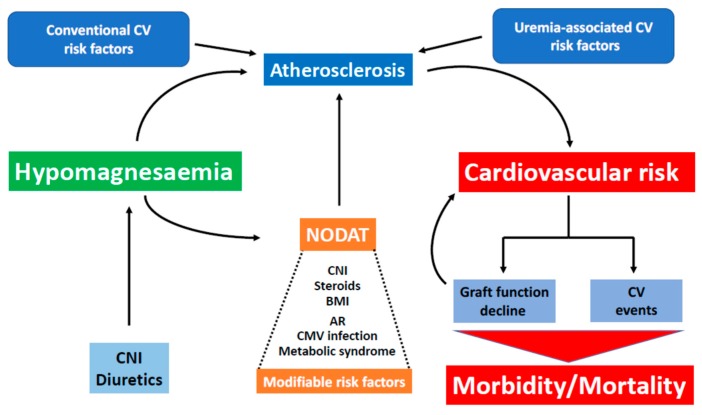
Deleterious effects of hypomagnesaemia after kidney transplantation. Several studies support that posttransplant hypomagnesaemia increases cardiovascular (CV) risk by increasing the risk of post-transplant diabetes mellitus (PTDM) development and by favoring accelerated atherosclerosis, along with other more conventional risk factors. We suggest that hypomagnesaemia correction soon after kidney transplantation may allow to decrease CV risk and result in less CV-related morbidity and mortality in kidney transplant recipients.

Despite relying on retrospective studies, a body of evidence links hypomagnesemia to PTDM and cardiovascular risk in kidney transplant patients (Figure 2). Given the frequency of PTDM and its relationship with cardiovascular risk, correcting hypomagnesemia soon after transplantation could translate into a significant decrease in vascular disease, which today is the primary cause of death in kidney transplant recipients. Thus, prospective studies to evaluate the impact of hypomagnesemia correction after kidney transplantation, as well as the best ways of achieving correction are needed.

## Abbreviations

AUC	Area under the curve
CKD	Chronic kidney disease
CNI	Calcineurin inhibitors
ESRD	End-stage renal disease
HD	Hemodialysis
HOMA_IR	Homeostasis model assessment-estimated insulin-resistance index
Mg	Magnesium
PD	Peritoneal dialysis
PTDM	Post-transplant diabetes mellitus
TRPM6	Transient Receptor Potential Melastatin 6
